# Exogenous monoterpenes mitigate H_2_O_2_-induced lipid damage but do not attenuate photosynthetic decline during water deficit in tomato

**DOI:** 10.1093/jxb/erad219

**Published:** 2023-06-05

**Authors:** Hao Zhou, Kirsti Ashworth, Ian C Dodd

**Affiliations:** Lancaster Environment Centre, Lancaster University, Library Avenue, Lancaster LA1 4YQ, UK; Lancaster Environment Centre, Lancaster University, Library Avenue, Lancaster LA1 4YQ, UK; Lancaster Environment Centre, Lancaster University, Library Avenue, Lancaster LA1 4YQ, UK; University of Birmingham, UK

**Keywords:** Ascorbic peroxidase, malondialdehyde, oxidative stress, photosynthetic efficiency, *Solanum lycopersicum*, superoxide dismutase

## Abstract

Although monoterpenes are suggested to mediate oxidative status, their role in abiotic stress responses is currently unclear. Here, a foliar spray of monoterpenes increased antioxidant capacity and decreased oxidative stress of *Solanum lycopersicum* under water deficit stress. The foliar content of monoterpenes increased with spray concentration indicating foliar uptake of exogenous monoterpenes. Exogenous monoterpene application substantially decreased foliar accumulation of hydrogen peroxide (H_2_O_2_) and lipid peroxidation (malondialdehyde). However, it appears that monoterpenes prevent the accumulation of reactive oxygen species rather than mitigating subsequent reactive oxygen species-induced damage. Low spray concentration (1.25 mM) proved most effective in decreasing oxidative stress but did not up-regulate the activity of key antioxidant enzymes (superoxide dismutase and ascorbate peroxidase) even though higher (2.5 and 5 mM) spray concentrations did, suggesting a complex role for monoterpenes in mediating antioxidant processes. Furthermore, soil drying caused similar photosynthetic limitations in all plants irrespective of monoterpene treatments, apparently driven by strong reductions in stomatal conductance as photosystem II efficiency only decreased in very dry soil. We suggest that exogenous monoterpenes may mitigate drought-induced oxidative stress by direct quenching and/or up-regulating endogenous antioxidative processes. The protective properties of specific monoterpenes and endogenous antioxidants require further investigation.

## Teaser

Exogenous monoterpenes applied as a foliar spray to tomato plants under water deficit stress ameliorated drought-induced oxidative stress (H_2_O_2_content) and damage (lipid peroxidation), but not stomatal limitation of photosynthesis.

## Introduction

Biogenic volatile organic compounds (BVOCs), specifically terpenes, enhance plant resilience to abiotic stresses such as high temperature and soil drying (Peñuelas and Llusià, 2003). The diverse group of compounds known collectively as monoterpenes (MTs, C_10_H_16_) are the second most important BVOC by global emission rate, surpassed only by isoprene. Their biosynthesis via the methylerythritol phosphate (MEP) pathway (Peñuelas and Staudt, 2010) is affected by environmental conditions such as light, temperature and CO_2_ level (Sharkey *et al.*, 2007), closely related to photosynthetic activity, and regulated by carbon and energy supply. Nevertheless, when environmental stresses limit photosynthesis, biosynthesis of certain terpenes can be maintained, likely by using alternative carbon sources (Brilli *et al.*, 2007) such as starch breakdown (Karl *et al.*, 2002), cytosolic carbon supply (Fortunati *et al.*, 2008), or precursors such as cytosolic pyruvate (Rosenstiel *et al.*, 2003). While their production and emission are constitutive, abiotic stresses can enhance terpene production dependent on the severity of stress (Peñuelas and Staudt, 2010). Environmental stress can also alter the composition of MTs emitted, likely because different compounds diffuse through the stomata at different rates (Harley, 2013), but possibly also depending on the physiochemical properties of these compounds and cellular lipid structure (Niinemets *et al.*, 2004; Noe *et al.*, 2006).

Terpene biosynthesis and emission are energetically expensive and must therefore provide a net benefit. Under abiotic stresses, plants that sustain terpene production and emissions maintain key functionality (Peñuelas and Llusià, 2003; Velikova and Loreto, 2005; Sharkey *et al.*, 2007), enabling plants to cope with extreme environmental conditions such as heatwaves and drought (Peñuelas and Llusià, 2003). Isoprene maintains relatively high photosynthesis and electron transport rate, decreases oxidative status, and enhances recovery after stress in plants exposed to high temperatures (>45 ℃) (Velikova and Loreto, 2005) and drought (Monson *et al.*, 2021). More recently, some MTs (e.g. α- and β-pinene) have been shown to exhibit isoprene-like functionality and are increasingly associated with stress defences of plants exposed to high temperatures (Copolovici *et al.*, 2005; Zuo *et al.*, 2017) and ozone (Loreto *et al.*, 2004). Exogenous MT treatments enhance thermotolerance by maintaining photosynthetic efficiency and decreasing photosynthetic limitation by providing potent antioxidant protection of cell membranes (Loreto *et al.*, 1998; Delfine *et al*., 2000; Peñuelas and Llusià, 2002). However, these protective effects vary between MT compounds (Copolovici *et al.*, 2005) and there is currently no direct evidence that MTs provide similar protection against water deficit.

Global warming is expected to increase the frequency, intensity, and duration of soil drying events (Caretta *et al.*, 2022), increasing drought stress in plants. When plant water losses exceed root water uptake, cellular turgor and leaf water potential (Ψ_leaf_) decrease, thereby suppressing physiology, growth and development (Lambers *et al.*, 2008). Plants use various signalling processes to maintain leaf water potential during early drought stages by decreasing stomatal conductance to water vapour (Huntenburg *et al.*, 2022). Stomatal closure limits transpiration rate, intercellular CO_2_, and net photosynthetic rate (Chaves *et al.*, 2003) but the involvement of MTs in these processes is not clear (Xu *et al.*, 2022).

Leaf water deficit disrupts the transfer of photon energy during photosynthesis. Excess electrons accumulate around the photosystems causing the photoreduction of oxygen molecules (O_2_, the Mehler reaction), inevitably producing large quantities of reactive oxygen species (ROS), including singlet oxygen, superoxide (•O_2_^−^), the hydroxyl radical (HO•), and hydrogen peroxide (H_2_O_2_), which are phytotoxic when accumulated in excess (Asada, 2006). Prolonged stress conditions cause increasing photooxidation (Pintó-Marijuan and Munné-Bosch, 2014) and rapid lipid peroxidation, thereby damaging cellular structures and the photosynthetic apparatus (Smirnoff, 1993). However, plants have evolved a range of enzymatic and non-enzymatic antioxidant mechanisms to control ROS levels to minimize oxidative damage and maintain the redox balance (Das and Roychoudhury, 2014). The most important enzymatic antioxidation mechanism for photosystem II (PSII) is the water–water cycle of the Mehler reaction (Asada, 2006). Excess electron flux from the photosystems induces the photoreduction of O_2_ producing •O_2_^−^, which is reduced to H_2_O_2_ by superoxide dismutase (SOD) and is itself detoxified to H_2_O by the ascorbate peroxidase (APX)-catalysed ascorbate and monodehydroascorbate radical cycle, using ascorbic acid as a reducer. Ascorbate regeneration also provides an effective dissipation route for electron flow in photosystem I. The water–water cycle thus efficiently decreases ROS accumulation induced by excess photon energy (Asada, 1999; Miyake, 2010).

Specific terpenes can provide an alternative source of antioxidants for plants (Vickers *et al.*, 2009a; Pollastri *et al.*, 2021). Isoprene- and MT-emitting or fumigated plants have lower ROS accumulation and lipid peroxidation under heat, ozone, and in the case of isoprene, drought stress (Loreto *et al.*, 2004; Velikova and Loreto, 2005; Vickers *et al.*, 2009b; Ryan *et al.*, 2014). Terpenes are thought to directly quench stress-induced ROS (Loreto *et al.*, 2001; Vickers *et al.*, 2009b) due to their chemical reducing properties (Graßmann, 2005), or act as a signalling molecules triggering systemic defences (Loreto and Schnitzler, 2010; Zuo *et al.*, 2019). However, MT-mediated ROS scavenging and antioxidative protection may depend on the availability of alternative endogenous antioxidant mechanisms such as photorespiration and ascorbate (Peñuelas and Llusià, 2002; Nogués *et al.*, 2015). Under high light intensity, terpenes work synergistically with other antioxidants to provide photoprotection (Brilli *et al.*, 2022), mitigating oxidative damage by quenching excess energy and thereby maintaining photosynthesis (Velikova *et al.*, 2008).

Monoterpenes appear to induce similar effects to isoprene, and their diversity and reducing potential may provide more targeted protection. Exogenous applications of terpinene and β-pinene restored antioxidant enzyme activity (i.e. SOD) and non-enzymatic antioxidants (i.e. carotenoids) under heat stress, possibly by up-regulating downstream reactions of the MEP pathway (Tian *et al.*, 2020). Other exogenous MTs conferred thermal protection to PSII (Loreto *et al.*, 1998; Delfine et al., 2000). Nevertheless, the antioxidant protection offered by MTs is not always accompanied by photosystem protection (Peñuelas and Llusià, 2002), and high MT concentrations (>2.5 mM) can directly cause oxidative stress and inhibit development (Ibrahim *et al.*, 2004; Singh *et al.*, 2006). It is not clear whether these responses to specific MTs also occur under water deficit conditions or how this relates to endogenous antioxidants.

To investigate whether MTs protect plants grown in drying soil, exogenous MTs were applied to tomato, a high MT-emitting species (Zhou *et al.*, 2022), and exposed to different irrigation treatments. We hypothesized that exogenous MTs maintain PSII photosynthetic activities under water deficit by increasing foliar antioxidative capacity, thus decreasing oxidative stress and damage to plants, and that this protective effect would be proportional to the concentration of MTs applied.

## Materials and methods

### Plant materials and growth

Tomato (*Solanum lycopersicum* cv. Ailsa Craig) seeds were germinated in John Innes No. 2 compost (Westland Horticulture Ltd, Tyrone, UK) in seed trays (5 × 4.8 × 5 cm cells). Three weeks after sowing, 144 uniform seedlings were selected and transplanted to 2-litre plastic pots (top 14 cm, base 10.5 cm, depth 18.5 cm), filled with John Innes No. 2. Plants were numbered and randomly assigned to one of four 1-m^3^ semi-controlled growth chambers constructed with clear Perspex acrylic sheet, similar to those described by Stokes *et al.* (1993). Plants were grown for a further 4 weeks and rotated between chambers every week. At this stage, the sizes of the plants were relatively similar, ranging from 20 to 25 cm in height, with seven to eight leaves. Plants were then rotated within each chamber every other day and between chambers every 4 d. Plants were fed fortnightly with Miracle-Gro All Purpose Soluble Plant Food at a concentration of 2.5 ml l^−1^ of water, following the manufacturer’s recommendation (The Scotts Company Ltd, Godalming, UK).

Growth lamps (Powerstar HQI-BT, 600 W/D daylight, Osram, Munich, Germany) provided 400 ± 20 μmol photons m^−2^ s^−1^ photosynthetic photon flux density (PPFD) at the level of the sampled leaf for 12 h per day (07.00–19.00 h) throughout the experiment. Day:night temperatures and relative humidity were maintained at 22 °C:16 °C (±1.0 °C) and 40:60 (±10%), respectively, by pumping air through the chambers at a flow rate of 3.0 ± 0.2 m^−3^ min^−1^.

### Treatments

Three independent, factorial experiments were conducted, each with different watering regimes and MT applications across four growth chambers. Well-watered plants were irrigated twice a day (08.00 and 18.00 h) by replacing 100% of daily pot water loss. Water deficit plants were irrigated once a day (18.00 h) by replacing 25% of individual daily pot water loss (by evapotranspiration). The water deficit treatment commenced 7 weeks after sowing and was continued for 7 d until wilt for all three experiments. The evening before starting the water deficit treatment (at 18.30 h), the plants received a foliar spray of MT solution to both sides of all leaves to drip point. The spray was then similarly applied twice a day (at 08.30 and 18.30 h) for the duration of the experiment. In experiments 1 and 2 a 1.25 mM MT solution or 5 mM solution, respectively, was applied, while in experiment 3 a range (1.25, 2.5 and 5 mM) of MT solutions were applied. In all three experiments, control plants were sprayed with a 0 mM MT solution.

### Monoterpene solutions

The MT compounds included in the exogenous spray were selected based on the composition of MT emissions from well-watered *Solanum lycopersicum*. These were determined in previous experiments under the same growth conditions (following the method described in Zhou *et al.*, 2022) and therefore assumed to reflect the endogenous MTs of *Solanum lycopersicum* cv. Ailsa Crag.

MT solutions were prepared by dissolving 800 µl each of α-pinene, β-pinene, 3-carene, α-terpinene, α-phellandrene, *p*-cymene, limonene, γ-terpinene, and terpinolene (Sigma-Aldrich Ltd, Gillingham, UK) in 0.1% (v/v, 10 ml) methanol. Milli-Q water was then added to make up 1 litre of 5 mM MT solution. This solution was further diluted with Milli-Q water to make 2.5 mM and 1.25 mM solutions, as required. The control (0 mM) solution was prepared by adding 990 ml Milli-Q water to 10 ml methanol (0.1% v/v).

### Sampling

Sampling started from day 0, when the plants were well-watered, for baseline measurements, and was then carried out daily until day 7. Sampling was conducted before irrigation and spraying of water deficit treatments and at least 2 h after well-watered plants were irrigated and sprayed. The sampled plants were randomly selected, with the leaflets adjacent to the terminal leaflet on the newest fully developed leaf used for non-destructive physiological measurements, or for destructive measurements such as biochemical assays and leaf water status. Sampled leaflets measured 5.5–7.0 cm in length, 2.5–3.0 cm in width.

Two plants from each treatment were used for Li-Cor measurements (physiology) and then harvested for leaf water potential and biochemical analysis. One plant was used for destructive measurements only, and Li-Cor measurements were carried out on one further plant, which was not harvested and was measured daily throughout the experiment. A total of three physiological and biochemical replicates were sampled at each time point during each experiment, ultimately giving each variable at least six replicates at 1.25 mM and 5 mM, and three replicates at 2.5 mM per sampling time. Substrate moisture level was measured immediately after sampling using a soil moisture sensor (WET-2, Delta-T Devices Ltd, Cambridge, UK) inserted to a depth of ~8 cm from the top of the pot. Measurements and sampling were performed from 10.30 to 18.30 h, with the first replicates of each treatment completed in treatment order first, then the second replicate and so on. Each measurement technique is described below.

### Physiology

A LI-6400XT portable photosynthesis system (LI-COR Inc., Lincoln, NE, USA) with an integral Leaf Chamber Fluorometer (LCF 6400-40) measured leaf gas exchange and (light-adapted) chlorophyll fluorescence. The leaflet was clamped inside a 1 cm^2^ circular sampling cuvette, positioned to avoid the leaf vein. Airflow to the cuvette was set to 500 mmol s^−1^ to provide positive pressure, and CO_2_ was provided by a CO_2_ mixer (Li-Cor 6400-01）and kept at a constant 412 ppm in the cuvette. The cuvette environment was allowed to stabilize for 5–10 min before readings were logged, every 60 s for 20 min, to record net photosynthetic rate (*P*_n_; μmol m^−^² s^−^¹), transpiration rate (*T*_r_; mmol m^−^² s^−^¹), and stomatal conductance (*G*_s_; mol m^−^² s^−^¹) to understand the fundamental physiological processes of gas exchange and stomatal behaviour. Full detailed settings of the instrument are available in Supplementary Table S1. Maximum fluorescence under a saturating light flash (*F*_m_ʹ), photosynthetic steady-state fluorescence (*F*_s_ʹ) and minimum fluorescence (*F*_0_ʹ) during momentary darkness were also recorded. Operating (Φ_PSII_, Eq. 1) and maximum (*F*_v_ʹ*/F*_m_ʹ, Eq. 2) efficiency of PSII, and PSII efficiency factor (*F*_q_ʹ*/F*_v_ʹ, Eq. 3) were estimated as described by Murchie and Lawson (2013) to understand photosynthetic performance after light adaption:


$$\begin{array}{l} \Phi_{PSII}=\;\frac{F_{q}\prime }{F_{m}\prime }=\frac{F_{m}\prime -F_{s}\prime }{F_{m}\prime } \end{array}$$ (1)

$$\begin{array}{l} \frac{F_{v}\prime }{F_{m}\prime }=\;\frac{F_{m}\prime -F_{0}\prime }{F_{m}\prime } \end{array}$$ (2)

$$\begin{array}{l} PSII\;\;\;factor=\frac{F_{q}\prime }{F_{v}\prime }=\frac{F_{m}\prime -F_{s}\prime }{F_{m}\prime -F_{0}\prime } \end{array}$$ (3)

### Plant water status

Ψ_leaf_ (MPa) was measured using the leaf one node below the sampled leaf, using a pressure chamber as described by Boyer (1967). In brief, leaves were cut from the stem using a sharp razor blade, and inserted into the pressure chamber with the petiole protruding from the seal gasket. After sealing the chamber, the pressure was gradually increased at a rate of 0.01 MPa s^−1^ until water exuded from the cut surface, indicating the pressure inside the chamber was equal to that of the xylem, and Ψ_leaf_ was read from the chamber gauge.

### Biochemical analysis

The leaflet used for Li-Cor measurements and MT sampling and its corresponding compound leaflet were collected and cut into strips using a razor blade. The strips were placed into separate 2.0 ml Eppendorf tubes, flash-frozen in liquid nitrogen, and stored at −80 °C. Samples were subsequently used for biochemical analyses, as described below. Assays included foliar MT content and measures of oxidative status, which we define as the balance between ‘oxidative stress’ and ‘enzymatic antioxidative activity’.

In this study, ‘oxidative stress’ refers specifically to foliar hydrogen peroxide (H_2_O_2_) content. H_2_O_2_ is a reactive oxygen species (ROS) indicator that is stable and easy to measure and is often used as a proxy for foliar ROS level (Pintó-Marijuan and Munné-Bosch, 2014). ‘Enzymatic antioxidative activity’ was measured here as the activities per unit of protein of superoxide dismutase and ascorbate peroxidase, which are routinely sampled chloroplast-related enzymes (Das and Roychoudhury, 2014). In addition, we use malondialdehyde (MDA) equivalents as a measure of ‘oxidative damage to lipids’. MDA is a reactive electrophilic species formed from lipid peroxidation, is generally correlated with oxidative stress, and is easy to detect (Hodges *et al.*, 1999).

#### Leaf monoterpene content

Frozen leaf material was freeze-dried for 48 h and subsequently ground into fine powder using a Mixer Mill MM 200. Approximately 30 mg of the ground dry leaf material was transferred to a pre-weighed 10 ml Falcon tube, which was then re-weighed to determine the exact weight of samples. Samples were extracted with 10 ml hexane via ultrasonication at 45 kHz for 1 h, followed by a shaking incubation at −4 °C overnight. The tubes were then centrifuged at 15 000 *g* for 10 min at −4 °C. The supernatants were transferred to another 10 ml Falcon tube and concentrated to a volume of less than 0.5 ml, hexane was added to achieve a final volume of 1 ml, and 0.5 ml aliquots were transferred to amber GC vials for MT analysis via gas chromatography–flame ionization detection.

The analysis was performed using an Agilent 7820A gas chromatograph system equipped with an HP-5 non-polar capillary column (30 m×0.32 mm×0.25 µm). Hydrogen was used as the carrier gas at constant flow of 1.5 ml min^−1^. The temperature of injection was 250 °C and injection volume was 1 µl using a split ratio of 1:10 with a split flow of 15 ml min^−1^. The oven temperature was initially held at 50 °C for 1 min, then elevated at a rate of 10 °C min^−1^ to 70 °C where it was held for 2 min. The temperature was then increased at a rate of 2 °C min^−1^ to 76 °C and held for 1.2 min. The oven was finally heated at 30 °C min^−1^ to 250 °C where it was kept for 2 min, giving a total run of 17 min. The temperature of the flame ionization detector was 300 °C with an air flow of 300 ml min^−1^, H_2_ flow of 30 ml min^−1^, and N_2_ flow of 30 ml min^−1^.

Peaks were identified by comparing the retention times (±5%) with standard compounds, for which the supernatant was replaced with 0.5 ml of a solution with the same composition as the spray solution. A calibration curve for quantification was constructed using a range of concentrations of standards (0.5, 1, 2.5, 5, 10, 20, 40, 50, 80, 100 µg ml^−1^). One duplicate was taken for every three samples to ensure the reproducibility of the analysis. Sample compounds not included in the standards were numbered with an MT prefix (e.g. MT1) and quantified using the calibration curve of α-pinene. Data acquisition, identification, and quantification were performed using OpenLAB CDS ChemStation (firmware revision: A.01.18.003; software driver version: 6.03.091). Leaf MT content was then calculated based on the dry weight of samples and expressed as mg g ^−1^ dry weight.

#### Reactive oxygen species and malondialdehyde

Aliquots of 100 mg and 40 mg fresh weight of frozen leaves were used for H_2_O_2_ and MDA assays, respectively. For homogenization and extraction, leaf materials were first ground using a pestle and mortar in a liquid nitrogen bath, then transferred to a 2 ml Eppendorf tube containing 0.1% (w/v) trichloroacetic acid (TCA), and vortexed. Subsequently, samples were homogenized in precooled tube blocks using a Mixler Mill (MM200, Retsch Ltd, Hope, UK). The homogenate was centrifuged at 12 000 *g* for 30 min at 4 °C, and supernatant was transferred to another Eppendorf tube for further assays.

H_2_O_2_ was determined as described by Klassen *et al.* (1994) and Velikova *et al.* (2000). In brief, 0.4 ml of the supernatant, generated as described above, was added to 0.4 ml of 10 mM potassium phosphate buffer (pH 7.0) and 0.8 ml of 1 M potassium iodide (KI). The coloured reaction product of H_2_O_2_ with KI developed within 25 min. The absorbance of the supernatant at 360 nm was determined, after colour stabilization for at least 1 h, using a spectrophotometer (Ultrospec 2100 pro, Biochrom Ltd, Waterbeach, UK). A calibration curve was produced by replacing samples with 0.4 ml of H_2_O_2_ solutions (0, 1, 5, 10, 20, 40, 50, 80, and 100 µM) diluted from commercial H_2_O_2_ solution (9.8 M, Sigma-Aldrich). H_2_O_2_ content was calculated from a calibration curve of absorbances of H_2_O_2_ standard solutions.

MDA content was determined by the thiobarbituric acid-reactive substances (TBARS) assay (Ryan *et al.*, 2014). In brief, 0.5 ml of the supernatant, generated as described above, was mixed with 1.0 ml of 20% TCA containing 0.5% (w/v) thiobarbituric acid (TBA) and the mixture heated in a water bath for 30 min at 95 °C. The reaction was then immediately stopped in an ice bath and the mixture centrifuged at 10 000 *g* at 4 °C for 5 min. Supernatant absorbance was again determined using a spectrophotometer, here at two wavelengths (532 and 600 nm) to correct for non-specific turbidity. An absorption coefficient of 155 000 μM^−1^ cm^−1^ was used (Heath and Packer, 1968) to calculate the MDA equivalents content of the samples.

#### Enzymes and total protein

Frozen leaf material (200 mg) was ground to a fine powder in liquid nitrogen, and the powder homogenized in 1.2 ml ice-cold potassium phosphate extraction buffer (pH 7.8, containing 0.1 mM EDTA) in a 2 ml Eppendorf tube. Samples were centrifuged at 15 000 *g* for 20 min at 4 °C and the supernatant was collected. The pellet at the bottom of the tube was re-suspended in 0.8 ml extraction buffer and then centrifuged at 15 000 *g* for a further 15 min at 4 °C. The supernatants were combined as crude leaf enzyme extract and stored on ice, to measure SOD and APX activity based on the total protein content. These assays were only performed in experiment 3.

Total SOD activity was measured by determining the sample’s ability to inhibit the photochemical reduction of nitro-blue tetrazolium chloride (NBT) based on the methodology of Giannopolitis and Ries (1977) as modified by Weydert and Cullen (2010). In short, each 2 ml of reaction mixture contained 100 μl leaf extract, 50 mM phosphate buffer (pH 7.8, 2 mM EDTA), 9.9 mM l-methionine, 55 μM NBT, 0.025% (v/v) Triton X-100, and 1 mM riboflavin. The reaction with NBT was initiated under a lamp providing ~380 µmol m^−2^ s^−1^ PPFD for 10 min. One control and one blank, each without leaf extraction, were illuminated with samples or kept in the dark, respectively, for 10 min to correct for background absorbance. Absorbance was read at 560 nm and SOD activity in units of U mg^−1^ protein was defined as the amount of SOD required to inhibit 50% of NBT photoreduction compared with the control.

APX activity was analysed based on the protocol of Nakano and Asada (1981). Each 1 ml reaction mixture contained 100 μl leaf extract, 50 mM potassium phosphate buffer (pH 7.0), 5 mM ascorbate and 1 mM EDTA. A reaction was initiated by adding 1 mM H_2_O_2_ and absorbance immediately recorded at 290 nm for 3 min. APX activity was determined using the extinction coefficient of reduced ascorbate (2.8 mM^−1^ cm^−1^) and expressed as mmol ascorbate min^−1^ mg^−1^ protein.

Total protein content of each sample, only used to define enzyme activities, was quantified by the Bradford (1976) method using bovine serum albumin as a standard.

### Statistical analysis

All statistical analyses were conducted using R v4.1.0. A general linear model with univariate ANOVA was used to determine significant differences in all independent variables (physiology, biochemistry, and Ψ_leaf_), and two-way and three-way interactions between the main effects (water deficit×exogenous MTs or concentrations). The soil–leaf water relationship was built based on van Genuchten (1980) using the R function ‘fitsoilwater’ in the package ‘soilphysics’ (de Lima *et al.*, 2021). Regression lines were estimated using a linear model to interpret relationships between Ψ_leaf_, physiological, and biochemical responses of plants. Significant differences between physiological and biochemical variables with leaf water potential or each other, as well as their interactions were determined by ANCOVA. A post-hoc Tukey test with Bonferroni correction was used to compare PSII efficiency variables between and within treatments at different water deficit levels. In all analyses, *P*<0.05 denoted statistical significance.

## Results

### Exogenous monoterpenes do not affect soil/plant water status

**Fig. 1.**
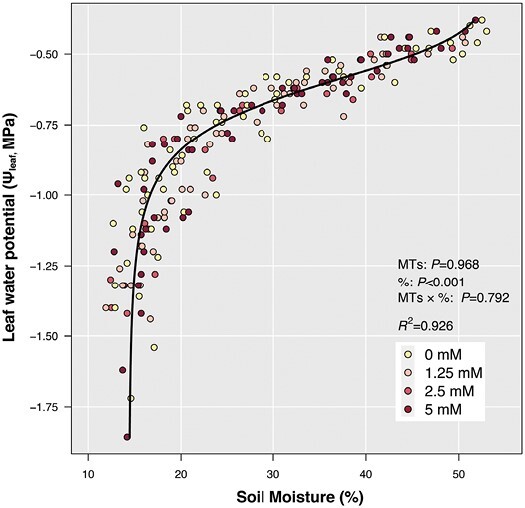
Leaf water potential (Ψ_leaf_) *vs* soil moisture for tomatoes treated with 0 mM (yellow), 1.25 mM (pink), 2.5 mM (red), and 5 mM (dark red) exogenous MT foliar spray. The response is described by the van Genuchten equation using R package ‘soilphysics’. Data from all three experiments are included. ANCOVA results (*P*-values reported) for the impact of exogenous MTs, soil moisture (%), and their interaction (MTs×%) are presented.

Initially, leaf water potential (Ψ_leaf_) declined relatively steadily from just over −0.5 MPa to approximately −0.75 MPa with soil moisture declining from 50% to ~21.7%, below which further soil drying decreased Ψ_leaf_ more sharply (Fig. 1) to less than −1.75 MPa and soil moisture reached ~15%. Neither experiment nor treatment (*P*>0.05) significantly affected this relationship, indicating that exogenous MTs did not affect Ψ_leaf_ response to soil drying. A three-way ANCOVA analysis showed no significant difference in plant responses to treatments between the three experiments.

### Exogenous monoterpenes increased foliar monoterpene content

**Fig. 2.**
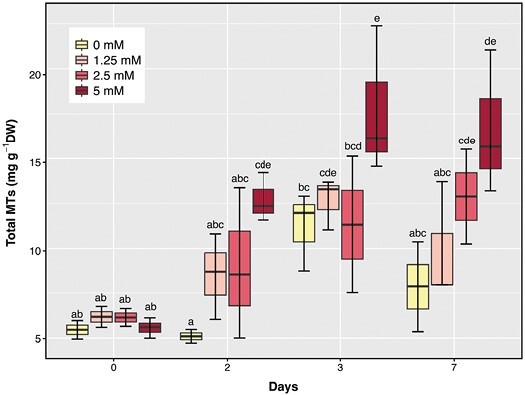
Total foliar MT content of plants treated with 0 mM (yellow), 1.25 mM (pink), 2.5 mM (red), and 5 mM (dark red) exogenous MT spray. Data from experiment 3 are included. ANOVA results with post-hoc test (significant difference is reported by letters) for the impact of exogenous MTs between treatments and days are reported.

Leaf MT content and composition were analysed on days 0 (well-watered), 2, 3, and 7 of the experiment to investigate MT content before during, and at the end of the drought regime. Total MT content increased by approximately 2.5-fold on day 3 in control (0 mM) plants as the soil dried, before decreasing by the end of the experiment (Fig. 2). Treating plants with exogenous MTs increased total foliar MT content from day 2 onwards, by 1.6-fold in plants treated with 1.25 mM and 2.5 mM and by 2.5-fold in plants treated with 5 mM. By the end of the experiment, exogenous MT application was required to sustain total MT content at the maximal levels induced by soil drying.

Analysing foliar MT composition revealed 24 compounds, including all of the exogenous MTs applied in the foliar spray and an additional 15 unidentified MTs within the range of retention times as the standard compounds in the calibration curve. The relative composition of foliar MTs remained nearly constant throughout the experiment. Supplementary Dataset S1 gives a complete list of compounds and their relative contributions and Supplementary Fig. S1 shows changes in composition over time for each treatment. α-Terpinene and MT11, an unidentified compound, were the most abundant in all treatments, each comprising nearly 20% of the total. Both 3-carene and terpinolene each contributed just over 15%. Following application of exogenous MTs, some compounds that were not present in the leaves prior to the start of spraying were also produced, and the proportion of some endogenous MTs was slightly reduced (by up to 4%, Supplementary Dataset S1; Supplementary Fig. S1). Therefore, exogenous MT not only increased leaf content of the applied MTs but also promoted the production of some endogenous MTs and inhibited the production of others.

### Exogenous monoterpenes mitigate oxidative response to water deficit

**Fig. 3.**
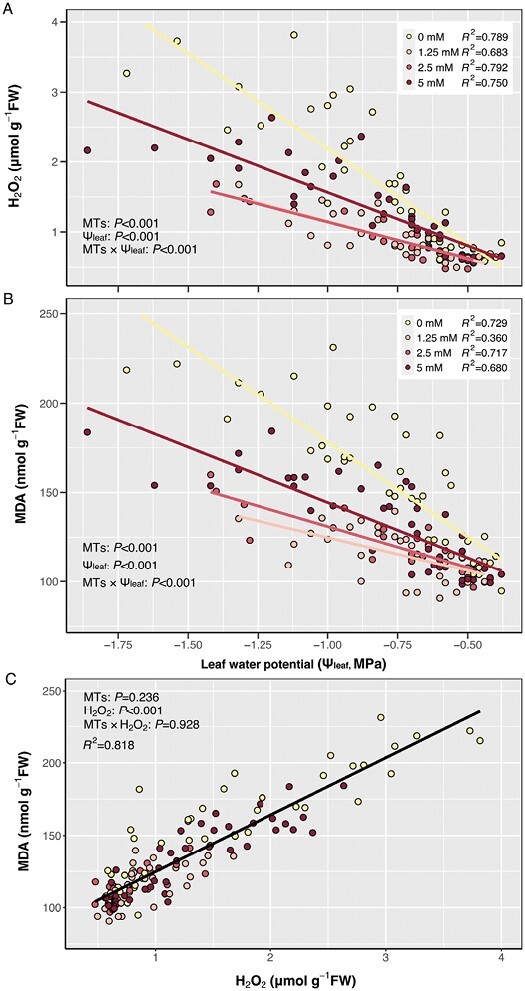
Relationships between foliar H_2_O_2_ (A) and MDA content (B) and leaf water potential (Ψ_leaf_, MPa), and between foliar H_2_O_2_ and MDA content (C) of plants treated with 0 mM (yellow), 1.25 mM (pink), 2.5 mM (red), and 5 mM (dark red) exogenous MT spray. Relationships are described by linear regression lines. Data from all three experiments are included. ANCOVA results (*P*-values reported) for the impact of exogenous MTs, H_2_O_2_, and leaf water potential (Ψ_leaf_) and their interaction (MTs×Ψ_leaf_/H_2_O_2_) are presented.

Oxidative stress, measured by foliar H_2_O_2_ content, increased linearly across all treatments as Ψ_leaf_ declined (Fig. 3A), but exogenous MTs attenuated the effect. In control (0 mM MT) plants, H_2_O_2_ content increased more than 6-fold at Ψ_leaf_ <−1.75 MPa. This was more than twice the final level of H_2_O_2_ in plants treated with 1.25 mM and 2.5 mM MT, which had the lowest H_2_O_2_ accumulation rate. Interestingly, plants treated with the highest MT concentration (5 mM) accumulated H_2_O_2_ at a rate intermediate between control and the lower treatment concentrations (1.25 mM and 2.5 mM), with a 4.7-fold increase. This higher MT concentration seemed less effective in mitigating oxidative stress.

Oxidative damage, measured as foliar MDA content, also increased linearly as Ψ_leaf_ declined (Fig. 3B), with MT concentration significantly (*P*<0.001) affecting MDA concentration. Again, control plants showed the greatest MDA accumulation (2.2-fold), while plants treated with 1.25 mM MT had the lowest levels (1.8-fold), with higher MT concentrations inducing an intermediate response. Oxidative damage (foliar MDA content) increased linearly with oxidative stress (foliar H_2_O_2_ content) similarly across all MT treatments (Fig. 3C). Thus, exogenous application of MTs appears not to interfere with ROS-induced lipid peroxidation, but rather mitigates oxidative damage by limiting H_2_O_2_ accumulation.

### High exogenous monoterpene concentrations induce antioxidant enzyme activity

**Fig. 4.**
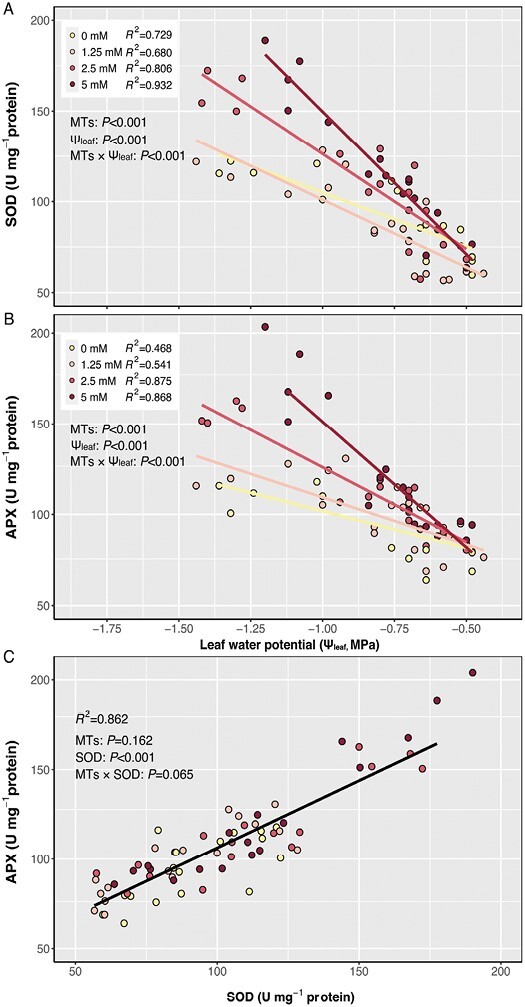
Relationships between foliar SOD (A) and APX activity (B) and leaf water potential, and between foliar SOD and APX activity (C) of plants treated with 0 mM (yellow), 1.25 mM (pink), 2.5 mM (red), and 5 mM (dark red) exogenous MT spray. Relationships are described by linear regression lines. Data are only available from experiment 3. ANCOVA results (*P*-values reported) for the impact of exogenous MTs and SOD with leaf water potential (Ψ_leaf_) and their interactions (MTs×Ψ_leaf_/SOD) are presented.

Activities of SOD and APX, the key enzymes involved in forming H_2_O_2_ from primary ROS and reducing H_2_O_2_ to H_2_O, respectively, increased linearly in all treatments as Ψ_leaf_ declined and H_2_O_2_ increased (Fig. 4A, B). At a given Ψ_leaf_, high MT concentrations promoted SOD and APX activities as indicated by significant interactions (*P*<0.001 for MTs×Ψ_leaf_). Plants treated with the lowest concentration (i.e. 1.25 mM) showed a similar response to control plants. Therefore, low concentrations of exogenous MTs did not affect enzymatic antioxidants, but high concentrations substantially increased antioxidant enzyme activities as leaf water status declined. All MT treatments showed a similar linear correlation (Fig. 4C, *P*>0.05) between SOD and APX activities, indicating that exogenous MTs do not appear to affect the detoxification of ROS to H_2_O.

### Exogenous monoterpenes do not affect photosystem II efficiency and gas exchange responses

**Fig. 5.**
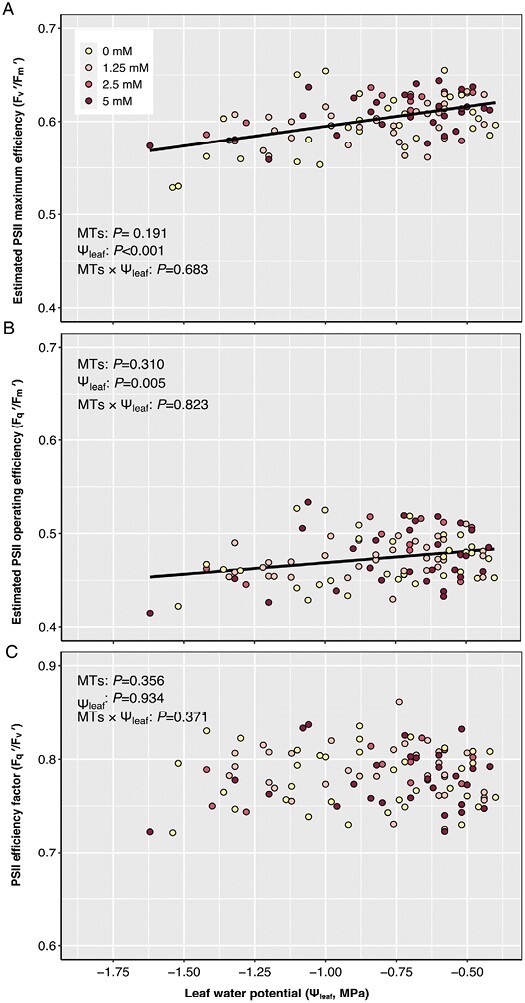
Estimated PSII operating efficiency (A), maximum efficiency (B), and efficiency factor (C) of plants under water deficit with 0 mM (yellow), 1.25 mM (pink), 2.5 mM (red), and 5 mM (dark red) exogenous MT spray. Data from all three experiments are included. ANCOVA results (*P*-values reported) for the impact of exogenous MTs and leaf water potential (Ψ_leaf_) on PSII with interactions are presented.

Leaf water deficit significantly decreased the estimated maximum efficiency (*F*_v_*ʹ/F*_m_*ʹ*, *P*<0.001) and operating efficiency (Φ_PSII_, *P*=0.005) of PSII photochemistry in light-adapted tomato leaves, with no effect of exogenous MT treatments (MTs×Ψ_leaf_*P*=0.68 and 0.82, Fig. 5A, B). Neither water deficit nor exogenous MTs affected the PSII efficiency factor (Fig. 5C), suggesting that the photochemistry of PSII remains unaffected by water deficit conditions.

**Fig. 6.**
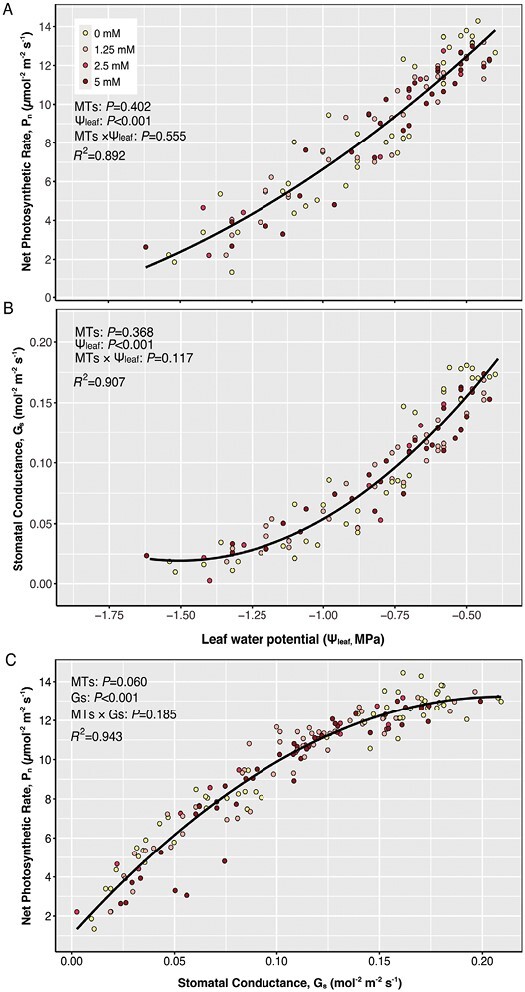
Relationships between stomatal conductance (*G*_s_) (A) and net photosynthetic rate (*P*_n_) (B) and leaf water potential, and between net photosynthetic rate (*P*_n_) and stomatal conductance (*G*_s_) (C) in plants exposed to 0 mM (yellow), 1.25 mM (pink), 2.5 mM (red), and 5 mM (dark red) exogenous MT spray. Data from all three experiments are included. ANCOVA results (*P*-values reported) for the impact of exogenous MTs with leaf water potential (Ψ_leaf_) and their interactions (MTs×Ψ_leaf_/Gs) are presented.

Leaf gas exchange (stomatal conductance, *G*_s_, and net photosynthetic rate, *P*_n_) decreased as Ψ_leaf_ decreased across all treatments (Fig. 6A, B), with no apparent effect of MT applications. Likewise, exogenous MT treatments did not affect the relationship between *G*_s_ and *P*_n_ (*P*=0.18, Fig. 6C), with the slope of this line indicating the intrinsic water use efficiency.

## Discussion

Foliar applications of a blend of exogenous MTs, similar in composition to those produced endogenously by tomato (Zhou *et al.*, 2022), increased total foliar MT content (Fig. 2) and decreased foliar H_2_O_2_ and MDA accumulation as the soil dried (Fig. 3). Although these MTs enhanced foliar enzymatic antioxidant capacity similarly to isoprene fumigation (Sharkey *et al.*, 2007), they had no effect on PSII efficiency or net photosynthesis of light adapted plants (Fig. 5, 6). Despite stimulating enzymatic antioxidant defences (SOD and APX activitie; Fig. 4), higher concentrations of exogenous MTs induced greater foliar oxidative stress than lower concentrations (Fig. 3), although less than control plants, suggesting a threshold MT for maximum protection against oxidative stress. To our knowledge, this is the first time that exogenous MTs have been shown to mitigate drought-induced oxidative stress.

Leaf water status declined with soil water content (Fig. 1) as in other studies that fully withheld irrigation from tomato (Živanović *et al.*, 2021), with reduced water transport from roots to leaves (Osakabe *et al.*, 2014) causing Ψ_leaf_ to decline (Lambers *et al.*, 2008). Exogenous MT applications did not affect this relationship. While partial stomatal closure acts to maintain Ψ_leaf_, exogenous MTs did not affect stomatal responses to leaf water deficit (Fig. 6B), even though increased MT concentrations have been correlated with stomatal closure in other species (Rai *et al.*, 2003; Sancho-Knapik *et al.*, 2017). Although plants exposed to drying soil received an irrigation volume equivalent to 25% of well-watered plant evapotranspiration, this was insufficient to maintain leaf water status (Ψ_leaf_ decreased by 0.23 MPa d^−1^ on average). In contrast, tomato plants grown under similar environmental conditions but receiving 50% evapotranspiration maintained a Ψ_leaf_ that averaged only 0.1 MPa lower than well-watered plants (Dodd, 2007). While many studies have investigated non-hydraulic signalling causing stomatal closure in tomato (e.g. Dodd, 2007; de Ollas *et al*., 2018), maintenance of Ψ_leaf_ as the soil dries, e.g. by growing plants in large soil volumes (Zhang and Davies, 1989), is most likely to discriminate chemical mechanisms regulating stomatal responses. Although exogenous MTs did not affect stomatal closure as the soil dried, this does not exclude the possibility that endogenous MT-related hormone interactions (e.g. MEP–ABA biosynthesis) affect stomatal regulation (Barta and Loreto, 2006). Future studies should measure and genetically manipulate endogenous MT production to investigate whether MTs affect stomatal behaviour in drying soil.

### Leaves absorb exogenous monoterpenes, which mediate endogenous monoterpene content

Leaf water deficit increased foliar MT content in all plants, suggesting endogenous MTs may mitigate oxidative stress and damage in tomato. However, average total foliar MT content in the exogenous MT treatment remained consistently higher than that of the control plants which received no MTs in the foliar spray (Fig. 2), suggesting significant uptake of exogenous MTs and accumulation in the leaves. Likewise, fumigation with a different mix of exogenous volatile MTs increased foliar MT content up to 5-fold (Delfine *et al.*, 2000) compared with 2.5-fold in our study, with these differences likely arising from the physiochemical properties of gaseous and aqueous states of the different MTs. These acquired MTs can be stored and even translocated within plant leaves, depending on the concentration and duration of MT application, implying that foliar uptake of MTs is a continuous process that is influenced by the concentrations used in spray.

The primary changes in foliar MT concentrations occurred in 3-carene, α-terpinene, terpinolene, and an unidentified compound designated as MT11 (Supplementary Dataset S1). These not only increased 2- to 3-fold in plants treated with exogenous MT treatments, but also in the untreated (control) plants in response to water deficit. Interestingly, other compounds such as MT1–4 and 12–15, which were not present in the exogenous MT spray or the well-watered plants on day 0, were also detected in all sprayed leaves during water deficit treatment. This suggests that oxidative stress induced by water deficit stimulates the biosynthesis and metabolism of certain endogenous MTs, and that exogenous MTs mediate this process. Other components of the foliar spray (e.g. *p*-cymene), tended to accumulate during the experiment, but to a much lesser extent. These observations support previous findings that both abiotic stresses (e.g. temperature and drought) and exogenous sources (Loreto *et al.*, 1998; Delfine *et al.*, 2000; Nogués *et al.*, 2015) change foliar MT concentrations and composition, but the magnitude of changes varies between compounds.

The storage and emission of endogenous MTs and uptake of exogenous MTs by plant leaves vary dramatically between compounds, dependent on the physiochemical characteristics of the individual compounds (Niinemets *et al.*, 2004; Copolovici *et al.*, 2005) and rate of direct uptake through the cuticle (Harley, 2013). For instance, α-terpinene and terpinolene exhibit greater solubility and are more conducive to intercellular accumulation than α-pinene and limonene, which are more volatile (Copolovici and Niinemets, 2005). Additionally, drought conditions directly (e.g. photosynthetic limitation) or indirectly (e.g. biosynthetic regulation) influence endogenous MT concentrations, which in turn affect leaf uptake driven by concentration gradients (Noe *et al.*, 2006, 2008). However, no information is available about the absolute uptake and consumption of exogenous MTs. For example, while α-terpinene and terpinolene increased in sampled leaves, the foliar content of several compounds, such as α-pinene and α-phellandrene, did not appear to change throughout the experiment. Whether this results from differential uptake, the loss of the more volatile MTs by rapid emission or use of the more reactive MTs for direct quenching of ozone, ROS, or free hydroxyl radicals is not clear. Further studies are required to determine the extent of uptake for specific MT components and their subsequent impact on biochemical responses, to understand how MTs acquired by the leaves are involved in drought responses.

### Exogenous monoterpenes prevent H_2_O_2_-mediated lipid peroxidation by decreasing H_2_O_2_ accumulation

Leaf water deficit results in oxidative stress and enhances production of cellular ROS that link signalling pathways and defence mechanisms using H_2_O_2_ as a secondary messenger (e.g. Cruz de Carvalho, 2008; Das and Roychoudhury, 2014). As leaf water status decreased, lipid peroxidation (MDA content) was linearly correlated with ROS accumulation (H_2_O_2_ content), as previously observed (Hasanuzzaman *et al.*, 2020; Liang *et al.*, 2020), with exogenous MTs not affecting this relationship. Nevertheless, applying exogenous MTs significantly decreased foliar H_2_O_2_ and hence MDA production under drought stress. Although terpenes can decrease damage from oxidative stress induced by various abiotic stresses (Loreto *et al.*, 2004; Vickers *et al.*, 2009b; Nogués *et al.*, 2015; Pollastri *et al.*, 2021), this is the first report that they can mitigate oxidative stress and damage in drought-exposed plants. However, foliar MT treatments at lower concentrations appeared more effective than the higher (5 mM) treatment. Possibly higher concentrations of some terpene compounds perturb the lipid fraction and disrupt protein properties of membranes, due to their low reactivity and high lipophilicity, thus causing lipid peroxidation and solute leakage, as observed with terpinolene (Singh *et al.*, 2009; Agus, 2021). Furthermore, some MTs such as α-pinene can induce ROS accumulation at concentrations >2.5 mM (Singh *et al.*, 2006).

A further question is how exactly MTs act to reduce oxidative stress and damage, with our investigations limited to lipid damage indicated by foliar H_2_O_2_ and MDA content. Other processes may also lead to photooxidative damage. For example, lipid peroxidation mediated by singlet oxygen (^1^O_2_), which is formed in the energy transfer between excited chlorophyll and O_2_ when intercellular CO_2_ concentration is decreased by stomatal closure (as in Fig. 6) under drought conditions (Cruz de Carvalho, 2008), differs from hydroxide-mediated lipid peroxidation (Triantaphylidès *et al.*, 2008). Although terpenes may stabilize the lipid structure of organelle membranes such as thylakoid membranes (Loreto *et al.*, 2001), the linear relationship between H_2_O_2_ and MDA observed across all treatments (Fig. 3C) suggested that exogenous MTs did not stabilize lipid structures under oxidative stress. Instead, their protective effects were simply conferred by decreasing accumulation of H_2_O_2_ under water deficit conditions. The specific terpenes that provide oxidative protection under drought stress may do so by acting as: (i) antioxidants that directly scavenge free radicals and superoxide and/or (ii) messengers and indirect antioxidants that enhance signalling pathways and thence both enzymatic and non-enzymatic antioxidant processes (Zuo *et al.*, 2019; Pollastri *et al.*, 2021).

The antioxidative capacity of terpenes generally depends on their specific biochemical properties, in particular their reducing capacity (Graßmann, 2005). For example, the *in vitro* reducing power of phellandrene is approximately twice that of limonene (Lado *et al.*, 2004), while the *in vitro* hydroxyl radical reaction rate, a proxy of reducing power, of myrcene is nearly quadruple that of α-pinene (Atkinson and Arey, 2003; Atkinson *et al.*, 2006), suggesting greater efficacy in decreasing oxidative stress *in vivo*. However, different membrane permeability and uptake, which are determined by the physiochemical properties of MTs and thus their intercellular concentrations, may also result in differences in antioxidative capacity. Despite showing similar radical reaction rates, fumigation by α-pinene moderately restored the heat tolerance of oak leaves in which fosmidomycin had suspended MT biosynthesis, whereas α-terpineol did not. Copolovici *et al.* (2005) ascribe this to the higher volatility of α-pinene, enabling greater uptake. Since the specific antioxidant effects of different terpenes are so variable, we applied a mixture of nine compounds and cannot confirm whether the observed protective effects are universal or the result of certain individual MTs.

### Exogenous monoterpene concentrations differentially affect antioxidative mechanisms

Under oxidative stress, the increased accumulation of ROS stimulates antioxidant processes (such as the SOD and APX enzymes) that are essential to maintain oxidative homeostasis and optimize cell functions and activities (Cruz de Carvalho, 2008). Soil drying increased SOD and APX enzyme activity as H_2_O_2_ accumulated in all treatments (Fig. 3), but exogenous MTs showed dose-dependent effects. Although the 1.25 mM treatment had no detectable effect on the foliar total activity of these key enzymes, higher concentrations (2.5 mM and 5 mM) promoted SOD and APX activity. Applying MTs at low concentration (1.25 mM) may diminish enzyme antioxidant effects by directly acting as an antioxidant or synergistically working with other antioxidants, thereby inhibiting or delaying the activation of endogenous antioxidant defences such as SOD- and APX-mediated activities.

Nevertheless, the oxidative status was not balanced by promoted enzymatic antioxidative activity. Indeed, higher MT treatments (2.5 mM and 5 mM) seemed to impose oxidative stress via unknown processes. This resulted in higher foliar H_2_O_2_ and MDA content than in the 1.25 mM treatments, although still lower than the control. In turn, this up-regulated SOD and APX antioxidant enzyme activities to a greater extent in 2.5 mM and 5 mM treatments. While up-regulating SOD converted highly toxic superoxide radicals to the less toxic H_2_O_2_ more rapidly, a similar up-regulation of APX further detoxified excess H_2_O_2_ to water at a similar rate (Fig. 4C). This water–water cycle, which produces H_2_O_2_ from superoxide in the chloroplast, also provides an alternative pool for electrons from the photosystem, which are required for O_2_ photoreduction and ascorbate regeneration, thereby dissipating excess energy (photons) when photosynthesis is limited (Asada, 1999; Miyake, 2010). The Mehler–peroxidase reaction may account for up to 29% of photosynthetic electron flow to avoid over-reduction (Biehler and Fock, 1996). Therefore, higher MT concentrations may trigger enzymatic antioxidant defences to provide an additional energy dissipation process that works together with the reducing power of MTs (e.g. myrcene), thereby mitigating damage compared with the untreated (control) plants.

**Fig. 7.**
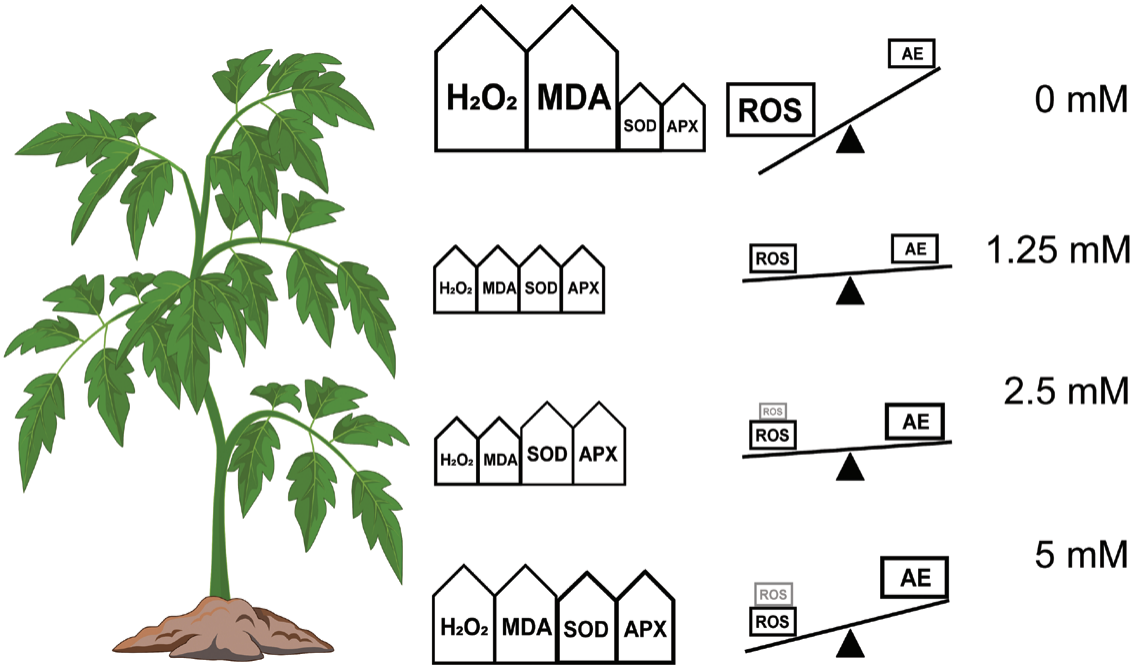
Illustrative sketch of exogenous MTs’ impact on foliar H_2_O_2_ (ROS, black frame), MDA content, and SOD and APX activities (antioxidative enzymes, AE). Grey ROS boxes indicate possible toxic effects of exogenous MTs. The rightmost column shows the effect on oxidative status and homeostasis. Based on concepts from Monaghan *et al.* (2009).

Figure 7 illustrates relationships between ROS (i.e. H_2_O_2_), antioxidant enzymes and oxidative status. Under non-stressed conditions, complex antioxidative defence pathways regulate ROS levels, preventing oxidative stress and damage (e.g. lipid peroxidation) to the plant and maintaining oxidative homeostasis, which results from the synergistic cooperation of antioxidant systems (Monaghan *et al.*, 2009). In contrast, leaf water deficit stimulates ROS production that exceeds the capacity of these systems including endogenous terpenoids, leading to oxidative stress and damage as evidenced here by the increased foliar H_2_O_2_ and MDA content of control plants. Applying 2.5 and 5 mM MT treatments up-regulated antioxidant enzyme activity, thereby alleviating oxidative stress and improving the redox state. Nevertheless, the oxidative status of plants in 2.5 and 5 mM treatments were not balanced suggesting alternative oxidative stress sources (indicated in grey in Fig. 7), possibly directly imposed by exogenous MTs. Notably, in the 1.25 mM treatment, ROS production and oxidative stress reached a near-equilibrium state despite no measurable change in antioxidant enzyme activity, implying that exogenous MTs and/or other antioxidant pathways contribute to oxidative homeostasis.

The antioxidative mechanisms in plant are complicated. Although this study measured only basic oxidative status and fundamental enzymatic antioxidative processes, our findings clearly show that MTs have an important role to play in mitigating the effects of drought stress. However, much remains to be done to fully elucidate the mechanisms involved. For example, non-photochemical quenching via the xanthophyll cycle (Cousins *et al.*, 2002) provides another primary chloroplastic energy dissipation pathway to prevent photoreduction when drought suppresses photosynthesis rate. This works synergistically with antioxidative enzymes, providing efficient photoprotection to plants (Beis and Patakas, 2012; Pintó-Marijuan and Munné-Bosch, 2014). It is worth noting that photorespiration also contributes to H_2_O_2_ production and tends to be more significant when drought stress decreases intercellular CO_2_ concentrations, interfering with redox balance and antioxidant status (Noctor *et al.*, 2002). Another detoxification mechanism of ROS involves non-enzymatic antioxidants, especially the scavenging of ^1^O_2_ by carotenoids (e.g. β-carotene). This is closely linked to both the chloroplastic photooxidative protection (Ramel *et al.*, 2012) and MEP synthesis pathway (Rodríguez-Concepción, 2010), and has been associated previously with both endogenous terpene production and exogenous MT application (Brilli *et al.*, 2022). Applying increased concentrations of terpinene and β-pinene, which are included in our foliar sprays, to heat-stressed plants increased endogenous carotenoid concentrations (Tian *et al.*, 2020). Although we focus on the key antioxidative enzymes SOD and APX (Fig. 4), the decreased oxidative stress and damage to lipids observed under the range of exogenous MT treatment concentrations applied here likely results from a range of oxidant–antioxidant reactions and energy dissipation pathways, which require further investigation.

### Monoterpene enhancement of antioxidative protection does not affect leaf gas exchange

Both natural isoprene and MT emitters, and plants fumigated with these terpenes, showed higher photosynthesis and PSII efficiency under oxidative stress (Delfine *et al.*, 2000; Loreto *et al.*, 2004; Vickers *et al.*, 2009b). Exogenous MTs helped to maintain chlorophyll fluorescence, photosynthetic efficiency and net photosynthesis under oxidative stress (Loreto *et al.*, 1998, 2004). Furthermore, heat-induced endogenous MTs have been associated with photosynthetic protection (Zuo *et al.*, 2017). While these studies suggest terpenes may protect the photosynthetic apparatus, foliar limonene application maintained chlorophyll fluorescence in carrot leaves at moderately elevated temperature, but did not sustain photosynthesis (Ibrahim *et al.*, 2004). Similarly, although exogenous MTs improved antioxidative capacity and mitigated drought-induced oxidative damage of tomato, they did not ameliorate declines in photosynthesis or maximum and operating photosynthetic efficiencies (Fig. 5, 6). Drought-induced decreases in stomatal conductance and intercellular CO_2_ concentration likely constrain photosynthesis independent of any non-stomatal responses. Whereas high exogenous MT (>2.5 mM) applications inhibited leaf gas exchange (Ibrahim *et al.*, 2004; Singh *et al.*, 2006, 2009), these concentrations did not affect tomato net photosynthesis (Fig. 6), probably because the overall oxidative status and damage were less than the control treatment.

It also appears that stomatal responses to leaf water deficit determined photosynthetic limitation as the soil dried. Nevertheless, PSII operating efficiency declined by ~12% as Ψ_leaf_ declined (Fig. 5B). Although statistically significant, this was much lower than the decreases of 35–45% in PSII efficiency when water was completely withheld from both tomato and tobacco plants (Mishra *et al.*, 2012; Ryan *et al.*, 2014). Less intense water deficit (Ѱ_leaf_≥−1.0 MPa) does not lead to long-term damage to the photosynthetic apparatus and less photochemical quenching, with photoinhibition and oxidative damage being preventing by up-regulation of non-photochemical quenching (Cousins *et al.*, 2002; Beis and Patakas, 2012). Since the antioxidative effect of exogenous MTs did not appear to affect the leaf PSII efficiency factor (Fig. 5C), it is likely that excess (photon) energy was not dissipated through photochemical quenching. When measuring chlorophyll fluorescence of dark-adapted plants, it is important in future studies to consider potential electron flow and energy dissipation routes that may affect the production and accumulation of reactive oxygen species and cellular oxidative homeostasis, as well as photosynthetic electron flow.

Plant emissions of BVOCs are thought to reflect the important role that these compounds play in plant resilience and tolerance to not only biotic but also abiotic stresses (Brilli *et al.*, 2019). Our findings support this and suggest that while exogenous MTs, such as limonene, are already used to increase plant resistance to pathogens (Simas *et al.*, 2017), they may have wider agricultural applications. The direct antioxidant properties of MTs and their possible interaction with other plant antioxidant mechanisms, shown here, suggest that applying MTs could mitigate damage from a wide range of environmental stresses. However, the specific benefits of MTs, both individually and in combination, need further investigation.

In conclusion, exogenously applied MTs were taken up by tomato leaves, increasing foliar antioxidative capacity as leaf water status declined. Specifically, exogenous MTs decreased oxidative stress (i.e. H_2_O_2_) thereby mitigating oxidative damage to lipid membranes. Nevertheless, leaf gas exchange and PSII efficiencies declined similarly in all plants as the soil dried, regardless of the concentration of MTs applied. Overall oxidative status depended on the MT concentration applied: 1.25 mM provided the best redox state (i.e. the least H_2_O_2_ accumulation and lipid peroxidation) likely because MTs directly scavenged ROS and synergistically worked with non-enzymatic antioxidants while higher MT concentrations (2.5 mM and 5 mM) further increased foliar H_2_O_2_ and MDA concentrations but also up-regulated enzyme (SOD and APX) activity, resulting in better oxidative status than that of untreated (control) plants. This suggests high doses of MTs may have been phytotoxic. Such dose-dependent effects suggest different response mechanisms with MTs also likely interacting with both antioxidants and energy dissipation pathways. These complex relationships and interactions between these various mechanisms require further investigation.

## Supplementary data

The following supplementary data are available at *JXB* online.

Fig. S1. Heatmap of total foliar MT content of plants treated with 0, 1.25, 2.5, and 5 mM exogenous MT spray by days.

Table S1. LI-6400XT specifications.

Dataset S1. A complete list of foliar monoterpene content (with standard deviation) of each compound and relative percentage of four treatments by days.

erad219_suppl_Supplementary_Figure_S1_Table_S1

erad219_suppl_Supplementary_Dataset_S1

## Data Availability

The data supporting the findings of this study are available within the paper and within its supplementary data published online.
