# Supplementary material for: Exogenous monoterpenes mitigate H2O2-induced lipid damage but do not attenuate photosynthetic decline during water deficit in tomato
Source: J Exp Bot. 2023 Jun 5;74(17):5327–40. doi: 10.1093/jxb/erad219 (PMC10498030; doi:10.1093/jxb/erad219)
Supplement: erad219_suppl_Supplementary_Figure_S1_Table_S1 [file erad219_suppl_supplementary_figure_s1_table_s1.pdf]

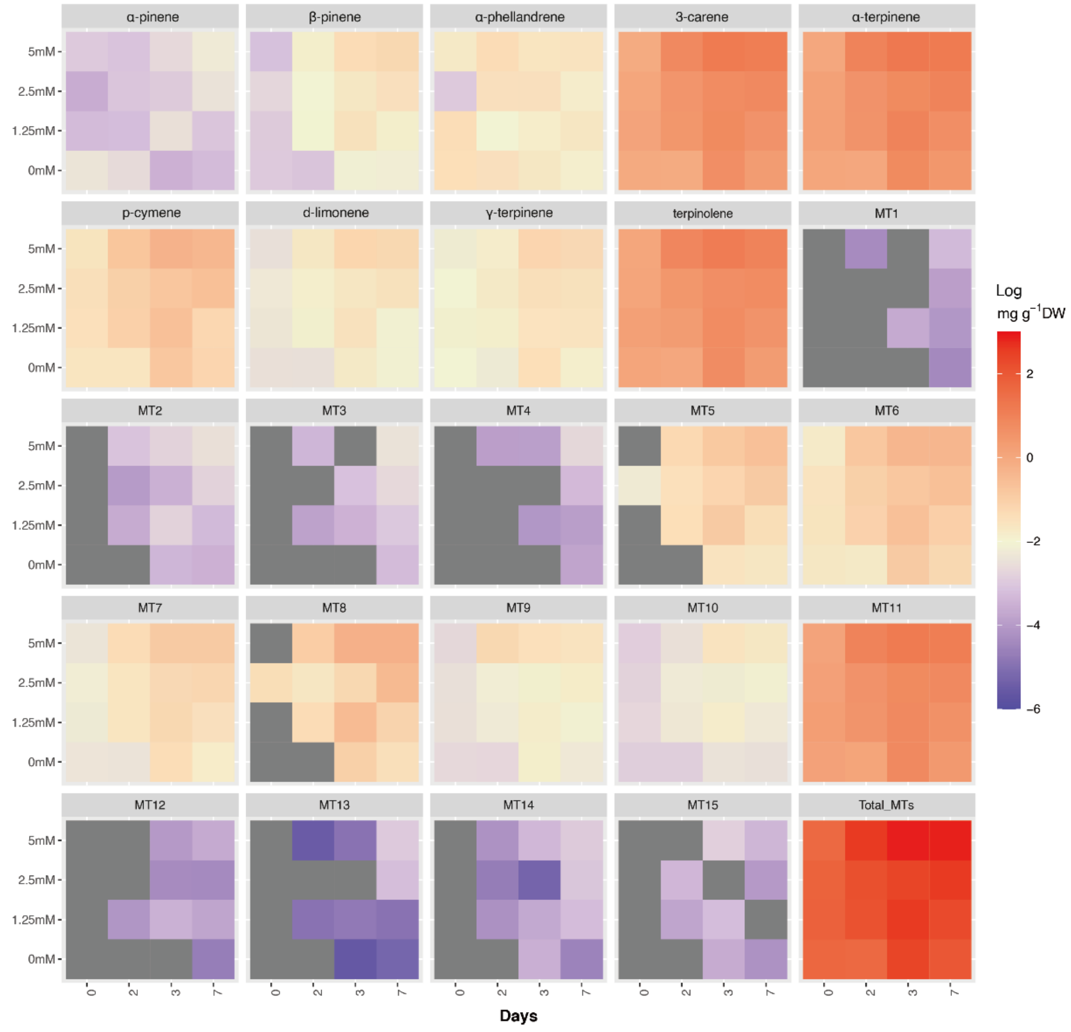

Figure S1. Heatmap of total foliar MT content of plants treated with 0 (yellow), 1.25 (pink), 2.5 (red) and 5 (dark red) mM exogenous MT spray by days. The values are presented on a log scale. Grey boxes indicate zero values. Data from experiment three are included.

Table S1. LI-6400XT specifications.

Settings	Values	
Air flow	500 mmol s^{-1}	
Light intensity	400 µmol photons m^{-2} s^{-1} (10% blue)	
Relative humidity	50 (± 10%)	
Reference CO_2	412 ppm	
Block temperature	22 ℃	
Leaf Chamber Fluorometer		
Measure	Intensity	2
	Modulation	20kHz
	Filter	1
	Gain	10
Flash	Type	Rectangular
	Duration	Adjusted to individual plants
	Intensity	8
	Modulation	20kHz
	Filter	50
Dark	Duration	8 sec
	Far-red intensity	8
	Pre-time	2 sec
	Post-time	4 sec
	Modulation	0.25kHz
	Filter	1
